# Supplementary material for: The impact of frailty and illness perceptions on quality of life among people living with HIV in Greece: A network analysis
Source: PLoS One. 2023 Nov 20;18(11):e0292787. doi: 10.1371/journal.pone.0292787 (PMC10659206; doi:10.1371/journal.pone.0292787)
Supplement: S1 Checklist — (PDF) [file pone.0292787.s001.pdf]

STROBE Statement—checklist of items that should be included in reports of observational studies

	Item No.	Recommendation	Page No.	Relevant text from manuscript
**Title and abstract**	1	(*a*) Indicate the study's design with a commonly used term in the title or the abstract	2	"This multicenter, cross-sectional study included…"
		(*b*) Provide in the abstract an informative and balanced summary of what was done and what was found	2	"**Methods** This multicentre … and consequences."
**Introduction**				
Background/rationale	2	Explain the scientific background and rationale for the investigation being reported	4-5	"Despite the scientific … result in a lower QoL."
Objectives	3	State specific objectives, including any prespecified hypotheses	5-6	"QoL, frailty and IP … QoL for people living with HIV
**Methods**				
Study design	4	Present key elements of study design early in the paper	7	"This study is a secondary analysis … Alexandroupoli, Patras)."
Setting	5	Describe the setting, locations, and relevant dates, including periods of recruitment, exposure, follow-up, and data collection	7	"This study is a secondary analysis … October 2022 to May 2023."
Participants	6	(*a*) *Cohort study*—Give the eligibility criteria, and the sources and methods of selection of participants. Describe methods of follow-up<br>*Case-control study*—Give the eligibility criteria, and the sources and methods of case ascertainment and control selection. Give the rationale for the choice of cases and controls<br>*Cross-sectional study*—Give the eligibility criteria, and the sources and methods of selection of participants	7	"Non-probability, consecutive sampling … no exclusion criteria for participation."
		(*b*) *Cohort study*—For matched studies, give matching criteria and number of exposed and unexposed<br>*Case-control study*—For matched studies, give matching criteria and the number of controls per case	-	Not applicable
Variables	7	Clearly define all outcomes, exposures, predictors, potential confounders, and effect modifiers.	7-8	"Frailty was assessed using …

		Give diagnostic criteria, if applicable		includes qualitative data."
Data sources/ measurement	8*	For each variable of interest, give sources of data and details of methods of assessment (measurement). Describe comparability of assessment methods if there is more than one group	7-8	"Frailty was assessed using … includes qualitative data."
Bias	9	Describe any efforts to address potential sources of bias	-	Not applicable because Non-probability, consecutive sampling was employed.
Study size	10	Explain how the study size was arrived at	7	"Non-probability, consecutive sampling was employed"

Continued on next page

Quantitative variables	11	Explain how quantitative variables were handled in the analyses. If applicable, describe which groupings were chosen and why	9-10	"In descriptive statistics, … qgraph and ggplot2 packages."
Statistical methods	12	(*a*) Describe all statistical methods, including those used to control for confounding	9-10	"In descriptive statistics, … qgraph and ggplot2 packages."
		(*b*) Describe any methods used to examine subgroups and interactions	-	We did not performed subgroups analysis.
		(*c*) Explain how missing data were addressed	10	"Any missing data were omitted."
		(*d*) *Cohort study*—If applicable, explain how loss to follow-up was addressed<br>*Case-control study*—If applicable, explain how matching of cases and controls was addressed<br>*Cross-sectional study*—If applicable, describe analytical methods taking account of sampling strategy	7	"Non-probability, consecutive sampling was employed"
		(*e*) Describe any sensitivity analyses	-	We did not perform sensitivity analyses.
**Results**				
Participants	13*	(a) Report numbers of individuals at each stage of study—eg numbers potentially eligible, examined for eligibility, confirmed eligible, included in the study, completing follow-up, and analysed	11-12	"Our sample included 477 participants … Table 3. Illness perceptions."
		(b) Give reasons for non-participation at each stage	-	Not applicable
		(c) Consider use of a flow diagram	-	Not applicable
Descriptive data	14*	(a) Give characteristics of study participants (eg demographic, clinical, social) and information on exposures and potential confounders	11-12	"Our sample included 477 participants … Table 3. Illness perceptions."
		(b) Indicate number of participants with missing data for each variable of interest	11-12	In tables 1-3 information for participants with full data for each variable of interest is provided.
		(c) *Cohort study*—Summarise follow-up time (eg, average and total amount)	-	Not applicable
Outcome data	15*	*Cohort study*—Report numbers of outcome events or summary measures over time	-	Not applicable
		Case-control study—Report numbers in each exposure category, or summary measures of exposure	-	Not applicable
		Cross-sectional study—Report numbers of outcome events or summary measures	11-12	"Our sample included 477 participants … Table 3. Illness perceptions."

Main results	16	(*a*) Give unadjusted estimates and, if applicable, confounder-adjusted estimates and their precision (eg, 95% confidence interval). Make clear which confounders were adjusted for and why they were included	13-20	"Correlations between measures … the different communities (Fig 3)."
		(*b*) Report category boundaries when continuous variables were categorized	-	Not applicable
		(*c*) If relevant, consider translating estimates of relative risk into absolute risk for a meaningful time period	-	Not applicable

Continued on next page

Other analyses	17	Report other analyses done—eg analyses of subgroups and interactions, and sensitivity analyses	19-20	"Network analysis … between the different communities (Fig 3)."
**Discussion**				
Key results	18	Summarise key results with reference to study objectives	21, 24	"The current study examined the impact … scientific area of HIV." "The interplay between QoL … optimize their health status."
Limitations	19	Discuss limitations of the study, taking into account sources of potential bias or imprecision. Discuss both direction and magnitude of any potential bias	24	"This study has several … determine their health status."
Interpretation	20	Give a cautious overall interpretation of results considering objectives, limitations, multiplicity of analyses, results from similar studies, and other relevant evidence	21-24	"Firstly, in the network … domain of their QoL."
Generalisability	21	Discuss the generalisability (external validity) of the study results	24-25	In the Limitations section, we discuss the limitations regarding our sample that readers have to take into consideration in order to assess the Conclusions of our study.
**Other information**				
Funding	22	Give the source of funding and the role of the funders for the present study and, if applicable, for the original study on which the present article is based	-	Based on the guidelines for authors of PLOS ONE, this information should be provided only in the submission platform.

*Give information separately for cases and controls in case-control studies and, if applicable, for exposed and unexposed groups in cohort and cross-sectional studies.

Note: An Explanation and Elaboration article discusses each checklist item and gives methodological background and published examples of transparent reporting. The STROBE checklist is best used in conjunction with this article (freely available on the Web sites of PLoS Medicine at http://www.plosmedicine.org/, Annals of Internal Medicine at http://www.annals.org/, and Epidemiology at http://www.epidem.com/). Information on the STROBE Initiative is available at www.strobe-statement.org.
