# Supplementary material for: The impact of frailty and illness perceptions on quality of life among people living with HIV in Greece: A network analysis
Source: PLoS One. 2023 Nov 20;18(11):e0292787. doi: 10.1371/journal.pone.0292787 (PMC10659206; doi:10.1371/journal.pone.0292787)
Supplement: S1 Appendix — (PDF) [file pone.0292787.s002.pdf]

1. **Weight loss** in last 12 months >4.5 kg

2. **Gait speed** on walking 4.5 meters

- Men:

For height <=173 cm, walking 4.5 meters in <=7 seconds.
For height >173 cm, walking 4.5 meters in <=6 seconds.

- Women:

For height <=159 cm, walking 4.5 meters in <=7 seconds.
For height >159 cm, walking 4.5 meters in <=6 seconds.

3. **Grip strength** (with Dynamometer)

- Men:

For BMI <=24, having grip strength <29 kg.
For BMI 24.1 to 28, having grip strength <30 kg.
For BMI >28, having grip strength <32 kg.

- Women:

For BMI <=23, having grip strength <17 kg.
For BMI 23.1 to 26, having grip strength <17.3 kg.
For BMI 26.1 to 29, having grip strength <18 kg.
For BMI >29, having grip strength <21 kg.

4. **Self-reported exhaustion** (derived from CES-D Depression Scale)

Patient rates either of the following 2 questions using a scale of 0-3 (0 = less than 1 day, 1 = 1-2 days, 2 = 3-4 days, 3 = most or all of the time) with a score of 2 or 3. In the last week, how many times did you feel that a) everything you did was an effort, and b) you could not get going?

- I felt that everything I did was an effort in the last week
- I could not get going in the last week

5. **Physical activity levels** were calculated with the use of the Greek version of the International Physical Activity Questionnaire (IPAQ-Gr).
