# Supplementary figures and images for: The impact of frailty and illness perceptions on quality of life among people living with HIV in Greece: A network analysis
Source: PLoS One. 2023 Nov 20;18(11):e0292787. doi: 10.1371/journal.pone.0292787 (PMC10659206; doi:10.1371/journal.pone.0292787)

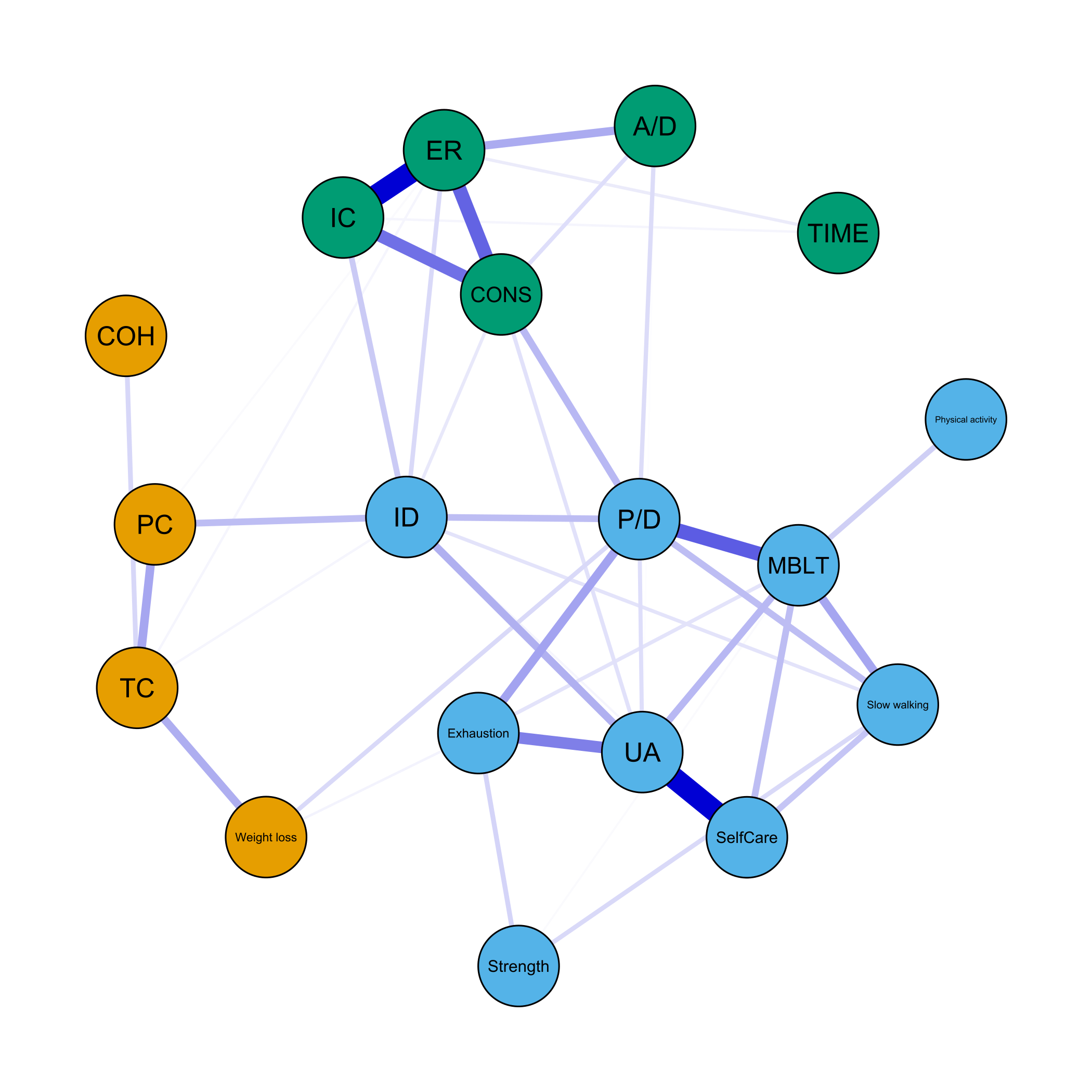

Supplement: S1 Fig — Abbreviations: A/D, Anxiety/Depression; UA, Usual activities; P/D, Pain/Discomfort; MBLT, Mobility; COH, Coherence; CONS, Consequences; ER, Emotional response; IC, Illness concern; ID, Identity; PC, Personal control; TC, Treatment control; TIME, Timeline. (TIF) [file pone.0292787.s003.tif]
